# High-Throughput MicroRNA Profiles of Permissive Madin-Darby Canine Kidney Cell Line Infected with Influenza B Viruses

**DOI:** 10.3390/v11110986

**Published:** 2019-10-25

**Authors:** Suthat Saengchoowong, Kritsada Khongnomnan, Witthaya Poomipak, Kesmanee Praianantathavorn, Yong Poovorawan, Qibo Zhang, Sunchai Payungporn

**Affiliations:** 1 Graduate Division, Faculty of Medicine, Chulalongkorn University, Bangkok 10330, Thailand; ssaengchoowong@gmail.com; 2 Department of Clinical Infection, Microbiology and Immunology, Institute of Infection and Global Health, University of Liverpool, Liverpool L69 7BE, UK; Qibo.Zhang@liv.ac.uk; 3 Department of Biochemistry, Faculty of Medicine, Chulalongkorn University, Bangkok 10330, Thailand; kritsada.kh@hotmail.com (K.K.); kesmanee1@hotmail.com (K.P.); 4 Chulalongkorn University Center of Excellence in Systems Biology, Research Affairs, Faculty of Medicine, Chulalongkorn University, Bangkok 10330, Thailand; nucler_13@hotmail.com; 5 Center of Excellence in Clinical Virology, Department of Pediatrics, Faculty of Medicine, Chulalongkorn University, Bangkok 10330, Thailand; yong.p@chula.ac.th

**Keywords:** microRNAs, influenza B viruses, Yamagata, Victoria, MDCK, next-generation sequencing, *Canis lupus familiaris*

## Abstract

Victoria and Yamagata lineages of influenza B viruses are globally circulating in seasonal epidemics. Madin–Darby canine kidney (MDCK) cells are permissive for viral isolation and vaccine manufacture. Nevertheless, the interplay between influenza B viruses and host microRNAs has not been investigated in this cell line. Therefore, the present study aims at high-throughput analysis of canine microRNA profile upon infection of influenza B viruses. Briefly, MDCK cells were infected with Victoria or Yamagata lineage at MOI of 0.01. After being harvested at 6, 12 and 24 h post infection, microRNAs were subjected to high-throughput sequencing based on MiSeq platform (Illumina). The results demonstrated that five microRNAs including cfa-miR-197, cfa-miR-215, cfa-miR361, cfa-miR-1841, and cfa-miR-1842 were overexpressed in both Victoria and Yamagata lineage infections. Interestingly, computational prediction showed that karyopherin alpha 6 (KPNA6) was targeted by cfa-miR-197 and cfa-miR-215. Moreover, the binding sites of both microRNAs were assessed by 3′-UTR reporter assay. The results showed that only cfa-miR-197 could bind to the target sites of KPNA6, leading to suppressing luciferase activity. Additionally, silencing of KPNA6 was confirmed by overexpression of cfa-miR-197. This study provides canine microRNA responses to seasonal influenza B viruses, suggesting that virus-mediated microRNAs might play crucial roles in host gene regulation.

## 1. Introduction

Seasonal influenza—a respiratory infectious disease with a high transmissibility rate and worldwide distribution—is caused by influenza A and B viruses [1]. Influenza A viruses have been extensively studied due to their association with high genetic variability and severe pandemics [2]. On the other hand, influenza B viruses were divided into two genetically and antigenically distinct strains, namely Victoria and Yamagata, in the 1980s [3]. However, molecular characterization of influenza B viruses has been little documented because a smaller impact of influenza B virus infection on public health was believed in the past [4]. Recently, epidemiological investigations have revealed a massive effect of influenza B on the mortality rate and the severity [5,6]. This evidence has highlighted the need to study the influenza B infections in order to understand the pathogenesis.

The isolation of viruses is an essential process needed to further study influenza viruses, e.g., molecular characterization, in vitro and in vivo investigations. Nowadays, African green monkey kidney (VERO) cell line as well as Madin–Darby canine kidney (MDCK) cell line is utilized as permissive cells for influenza viruses [7,8]. In addition to viral isolation, MDCK cell line has also been used for influenza vaccine manufacturing [9,10]. Nevertheless, mechanisms of influenza infections into permissive MDCK cells are little studied. More recently, there has been an increasing amount of evidence that microRNAs play critical roles in host–pathogen interactions [11,12]. MicroRNAs are a class of highly conserved noncoding single-stranded RNA approximately 18–25 nucleotides in length. It has been shown that microRNAs control gene expression post-transcriptionally by either translation inhibition or mRNA degradation [13]. Hence, the objective of the present study is to investigate microRNA profile of permissive MDCK cells upon influenza B virus infections.

## 2. Materials and Methods 

### 2.1. Cell Culture and Virus Infection

MDCK cells were seeded at 5 × 10^4^ cells per well in Dulbecco’s modified eagle medium (DMEM; GE Healthcare Life Sciences, Logan, UT, USA) containing 10% fetal bovine serum (Gibco, Logan, UT, USA) in 24-well plates under 5% CO_2_ at 37 °C for overnight. When the cells reached approximately 80% confluences, the media were removed. The cells were infected with mock influenza B virus Victoria lineage (B/Thailand/CU-B5522/2011), or Yamagata lineage (B/Massachusetts/2/2012) at the multiplicity of infection (MOI) of 0.01. After incubation with each viral suspension in overlay medium (DMEM supplemented with 0.2 µg/mL TPCK-treated trypsin (Sigma-Aldrich, St. Louis, MO, USA)) for 1 h, the viral suspensions were removed. The cells were washed with phosphate buffer saline (PBS; Merck Millipore, Darmstadt, Germany), and cultured with fresh infection medium (DMEM supplemented with 0.2% (*w*/*v*) bovine serum albumin (Sigma-Aldrich, St. Louis, MO, USA)), and 0.2 µg/mL TPCK-treated trypsin under 5% CO_2_ at 37 °C for 24 h.

### 2.2. MicroRNA and RNA Extraction

Cellular microRNAs were collected at 6, 12, and 24 h post infection (hpi). Briefly, the cells were washed with PBS and then dissociated with 0.05% trypsin/EDTA (Gibco, Grand Island, NY, USA). To isolate microRNA from the cell pellets, microRNA purification kit (Geneaid, New Taipei City, Taiwan) was used according to the manufacturer’s instruction. The concentration of microRNAs was quantified using Qubit fluorometer (Invitrogen, Singapore) with a Qubit^™^ microRNA assay kit (Invitrogen, Eugene, OR, USA). In addition, total RNAs were extracted using GenUP^™^ total RNA kit (Biotechrabbit, Berlin, Germany), and then quantified by NanoPhotometer^®^ (Implen, Munich, Germany).

### 2.3. Library Preparation and Next-Generation Sequencing

Purified microRNAs from the cells infected with the same viral strains and the same time-point were pooled together (*n* = 6). One-hundred nanogram of the microRNAs from each group was used to construct the libraries with different indexes according to a NEBNext^®^ Multiplex Small RNA Library Prep Set for Illumina^®^ (New England BioLabs, Ipswich, MA, USA). The concentration of DNA libraries was quantified by using KAPA Library Quantification Kits for Illumina^®^ Platform (Kapa Biosystems, Cape Town, South Africa). The DNA libraries were pooled together with equal concentration, and then single-end sequenced (50 cycles) on a MiSeq Benchtop Sequencer (Illumina, San Diego, CA, USA).

To analyze microRNA profile, MiSeq reporter software version 2.4 (Illumina, San Diego, CA, USA) was used for the primary analysis of sequencing data. In addition to the exclusion of low-quality reads (Q-score < 30), adaptor sequences were trimmed by the software. The passing filtered reads (Q-score ≥ 30) were aligned with canine genomic DNA (CanFam 3.1), mature and precursor canine microRNAs and contaminant RNAs. The sequencing reads matching to canine genomic DNA and contaminant RNA were discarded, whereas the reads matching to the microRNA database were considered as microRNAs. The microRNAs were identified and counted based on the number of reads matching to the miRBase [14]. As described in previous work [15], differential expression analysis was calculated in terms of fold change.

### 2.4. Reverse Transcription and Quantitative Polymerase Chain Reaction (RT-qPCR)

Prior to detecting the expression levels of candidate microRNAs, 100 µg of the microRNAs was polyuridylated by polyU polymerase (New England Biolabs). To generate cDNA, the microRNAs with polyU were then reverse transcribed with stem-loop (SL) poly A primers (5′-gtcgtatccagtgcagggtccgaggtattcgcactggatacgac-3′) [16]. On the other hand, total RNAs was reverse transcribed with oligo d(T)_16_ in order to detect the expression level of mRNA. RevertAid™ reverse transcriptase (Thermo Scientific, Vilnius, Lithuania) was used according to the manufacturer’s protocol. The qPCR reaction consisted of 5 μL of Luna^®^ Universal qPCR Master Mix (New England Biolab, Ipswich, MA, USA), 0.25 μL of each 10 μM forward and reverse primer, 1 μL of cDNA, and nuclease-free water was added to a final volume of 12 μL. The primer sequences and thermocycling conditions were available in Table 1. Real-time PCR amplification was conducted on Step One Plus™ Real-time PCR Systems (Applied Biosystems). The expression of canine RNA U6 Small Nuclear 2 (RNU6-2) and Glyceraldehyde-3-Phosphate Dehydrogenase (GAPDH) was measured as an internal control for microRNAs and mRNA expression, respectively. The results were analyzed using StepOne™ Software v.2.2 analysis (Foster City, CA, USA). The expression ratio was calculated by comparative ∆∆C_t_ method.

### 2.5. Classification of the Target Genes and In Silico Target Site Prediction

Following the targets of candidate microRNAs predicted by miRDB [17], the list of genes was classified according to biological processes by using PANTHER (Protein ANalysis THrough Evolutionary Relationships) classification system version 14.1 [18]. To predict the target sites, three web-based programs including TargetScan version 7.2 [19], miRDB [17], and RNAhybrid [20] were used on the basis of hybridization patterns between the microRNAs and their target mRNAs. Therefore, the criteria for the selection of microRNA targets based on effective hybridization patterns and minimum free energy (MFE) for base pairing less than −17.5 kcal/mol. 

### 2.6. Plasmid Construction

pSilencer 3.0-H1 (Ambion, Austin, TX, USA) and pmirGLO (Promega, Madison, WI, USA) were used as a vector backbone to produce microRNA expression vectors and reporter vectors, respectively. Each 10 μL of the top- and bottom-strand oligonucleotides (10 nM) was added with 5 μL of 5× rapid ligation buffer (Thermo Scientific, Vilnius, Lithuania), then denatured at 90 °C for 5 min, followed by annealing at 25 °C for 1 h (Table 2). Meanwhile, one μg of the pSilencer 3.0-H1 was cut with restriction enzymes *Bam*HI and *Hin*dIII (New England BioLabs, Ipswich, MA, USA), then incubated at 37 °C for 4 h. On the other hand, pmirGLO was cut with *Nhe*I and *Xho*I (New England BioLabs, Ipswich, MA, USA), followed by incubation at 37 °C for 4 h. For pmirGLO, the plasmids were treated with 1 µL of Antarctic phosphatase (New England BioLabs, Ipswich, MA, USA). After that, the annealed fragment was ligated into linearized pSilencer3.0-H1 or pmirGLO with T4 DNA ligase (Thermo Scientific, Vilnius, Lithuania), according to the manufacturer’s protocol. The plasmids were transformed into *E. coli* strain JM109 competent cells (RBC Bioscience, New Taipei City, Taiwan) by heat shock method. Ampicillin-resistant colonies were selected and propagated, followed by using HiYield^™^ Plasmid Mini Kit (RBC Bioscience, New Taipei City, Taiwan) for plasmid extraction. The concentration of each plasmid was measured by NanoPhotometer^®^ (Implen, Munich, Germany). To confirm the recombinant vectors, the nucleotide inserts were investigated by Sanger sequencing. 

### 2.7. Overexpression and Inhibition of microRNAs

MDCK cells were seeded into 24-well plates at 5 × 10^4^ cells per well overnight. For overexpression, the transfection of pSilencer_miR-197, pSilencer_miR-215, or pSilencer_Scramble was performed with Turbofect (Thermo Scientific, Vilnius, Lithuania), according to the manufacturers’ recommendation. On the other hand, miR-197 inhibitor (Ambion, Carlsbad, CA, USA) and negative control inhibitor (Dharmacon, Lafayette, Colorado, USA) were used for microRNA inhibition. The cells were transfected with Lipofectamine^®^ 2000 (Thermo Scientific, Vilnius, Lithuania), following the manufacturers’ protocol. After transfected, the cells were maintained at 37 °C in a humidified atmosphere containing 5% CO_2_ for 48 h. After incubation, microRNAs and total RNAs were collected and extracted in the Section 2.2.

### 2.8. Dual Luciferase Assay

For 3′-UTR reporter assay, MDCK cells were seeded at 10^4^ cells/well in media without antibiotic-antimycotic into 96 well-plates and incubated for 24 h. For transfection into each well, pmirGLO and pSlilencer were diluted with Opti-MEM (Gibco, Carlsbad, CA, USA), and then co-transfected into the MDCK cells by using Turbofect (Thermo Scientific, Vilnius, Lithuania), following the manufacturer’s instruction. The transfected cells were incubated under 5% CO_2_ at 37 °C for 48 h, and then harvested. The dual luciferase assay was conducted using Dual-Luciferase^®^ Reporter Assay System (Promega, Madison, WI) according to the manufacturer’s protocol. Briefly, the cells were washed with 100 µL of PBS and then added with 20 µL of Passive Lysis Buffer. The suspensions were then transferred into a Nunc^™^ F96 white plate (Thermo Scientific, Roskilde, Denmark). After that, 100 µL of luciferase assay reagent II (LARII) was added into each well. The emission of firefly luciferase activity at 560 nm was measured by Varioskan Flash Multimode (Thermo Scientific, USA) Before measuring *Renilla* luciferase activity at 480 nm, 100 µL of Stop and Glow reagent was added in order to stop firefly luciferase. The assay was done in triplicates. The relative luciferase activity was calculated using signal intensities of firefly luciferase divided by *Renilla* luciferase from a reporter vector. 

### 2.9. Statistical Analysis

The data were statistically analyzed and visualized by using GraphPad Prism version 8.1. The results were presented as the mean ± SD (standard deviation) of triplicates. Differences between two groups were analyzed using the Student’s unpaired *t*-test for gene expression and the Dunnett’s multiple comparisons test for luciferase activity. *p* values less than 0.05 (*p* < 0.05) were considered as statistically significant. 

## 3. Results

### 3.1. Differential Expression of Canine microRNAs upon Infection of Influenza B Viruses

**Figure 1:**
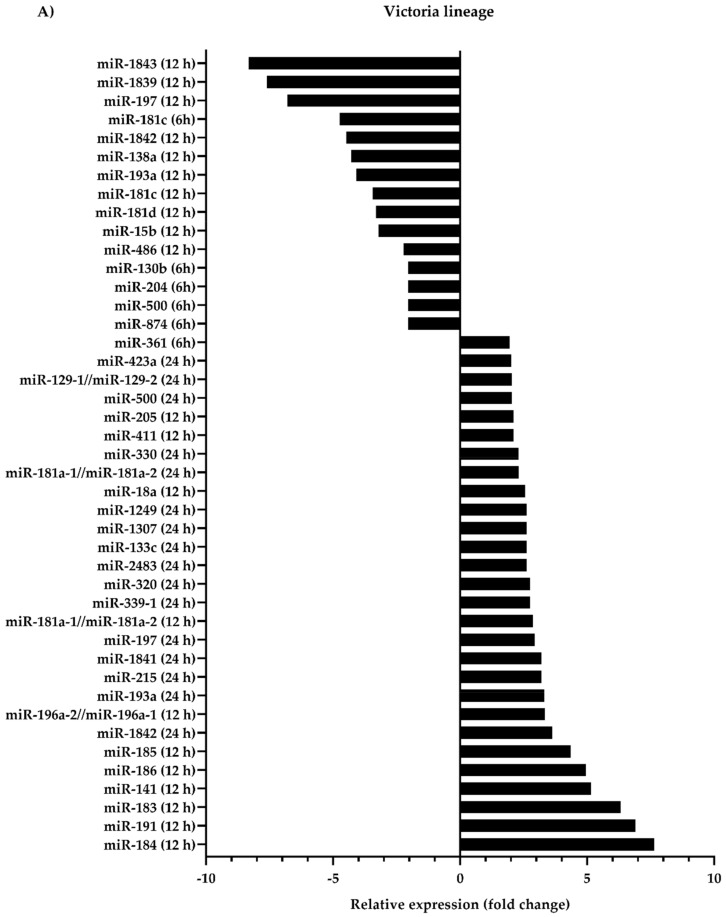

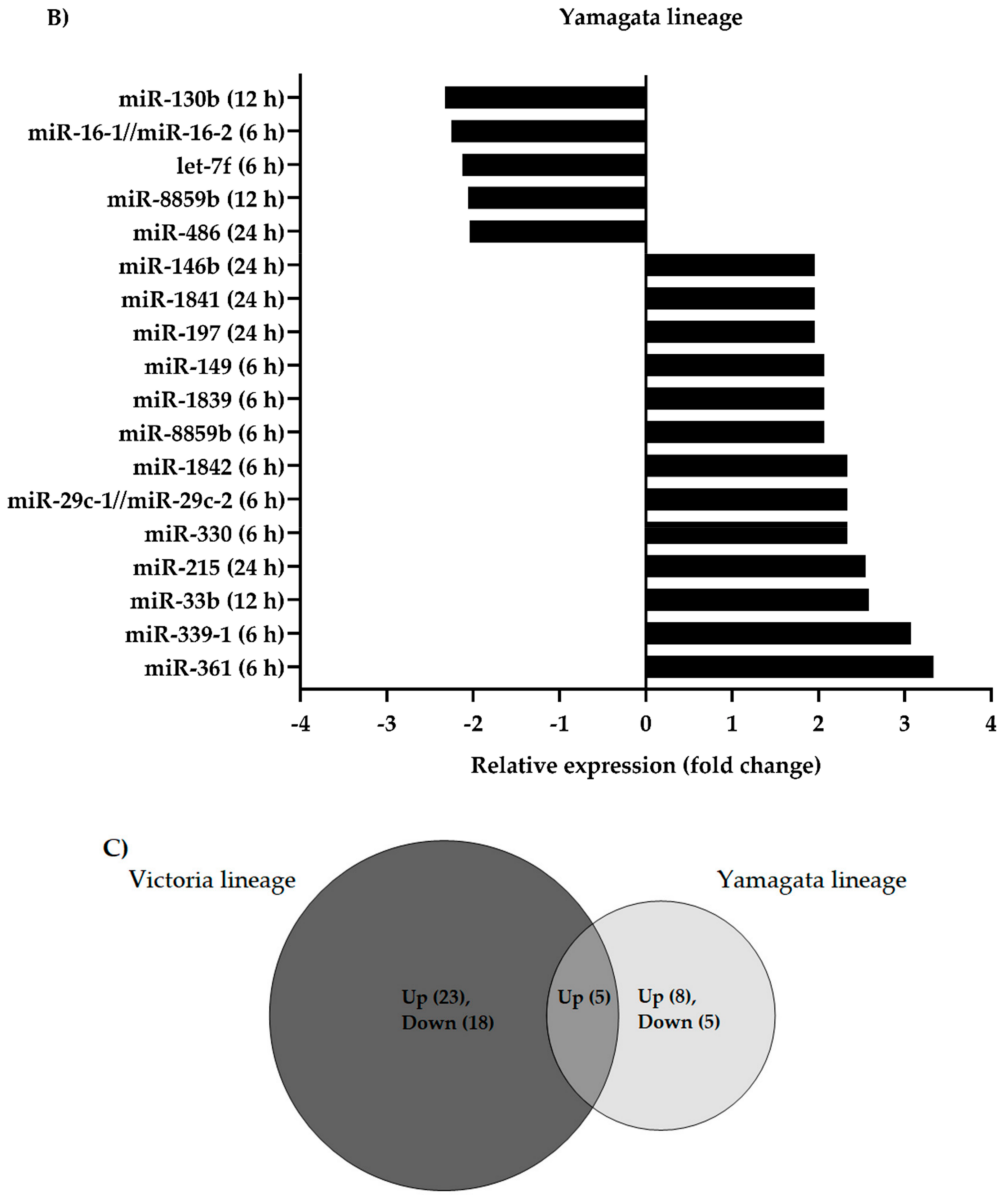
The miRNA expression profiles in each lineage of influenza B virus infection. Madin–Darby canine kidney (MDCK) cells were infected with mock, Victoria, or Yamagata lineage at multiplicity of infection (MOI) = 0.01, and collected at 6, 12, 24 hpi. (**A**) Forty-six microRNAs were dysregulated in the cells infected with Victoria lineage. (**B**) Eighteen microRNAs were in the cells infected with Yamagata lineage. (**C**) Venn–Euler diagram shows unique and overlapped microRNA expressions in Victoria and Yamagata lineages.

To identify canine microRNAs which were associated with influenza B virus infection, MDCK cells infected with Victoria lineage, Yamagata lineage, or mock were collected at 6, 12 and 24 h. Next-generation sequencing revealed the list of differentially expressed microRNAs with 2-fold or greater change in influenza B viruses-versus mock-infected cells. As shown in Figure 1A, 28 upregulated microRNAs and 15 downregulated microRNAs were presented in Victoria lineage infected MDCK cells. Among those microRNAs, cfa-miR-181a was upregulated at 12 and 24 hpi. While it decreased at 12 hpi, the expression of cfa-miR-197 was increased at 24 hpi. On the other hand, the overexpression of 13 microRNAs and the downexpression of 5 microRNAs were found in the cells infected with Yamagata lineage (Figure 1B). A comparative investigation of microRNAs in response to both lineages of influenza B viruses demonstrated that 38 and 13 unique microRNAs were dysregulated in the cells infected with Victoria and Yamagata lineage, respectively (Figure 1C). In contrast, five upregulated microRNAs including cfa-miR-197, cfa-miR-215, cfa-miR-361, cfa-miR-1841, and cfa-miR-1842 were overlapped between the two datasets.

### 3.2. Biological Classification of the Predicted Target Genes

The analysis of microRNAs’ response to influenza B virus infection showed that five microRNAs were commonly upregulated in the Victoria and Yamagata lineage-infected cells. The target genes of these microRNAs were predicted by miRDB database, and then classified using PANTHER. This study demonstrated that the categorization of the predicted target genes was based on biological processes such as biological adhesion (GO:0022610), biological phase (GO:0044848), biological regulation (GO:0065007), cell proliferation (GO:0008283), cellular component organization or biogenesis (GO:0071840), cellular process (GO:0009987), developmental process (GO:0032502), immune system process (GO:0002378), localization (GO:0051179), metabolic process (GO:0008152), multicellular organismal process (GO:0032501), pigmentation (GO:0043473), reproduction (GO:0000003), response to stimulus (GO:0050896), rhythmic process (GO:0048511), and signaling (GO:0023052). The number and the percentage of the target genes were summarized in Table 3. This finding showed that the major biological processes which were targeted by these five common microRNAs included cellular process, metabolic process, biological regulation, and localization.

### 3.3. Validation of cfa-miR-197 and cfa-miR-215

**Figure 2:**
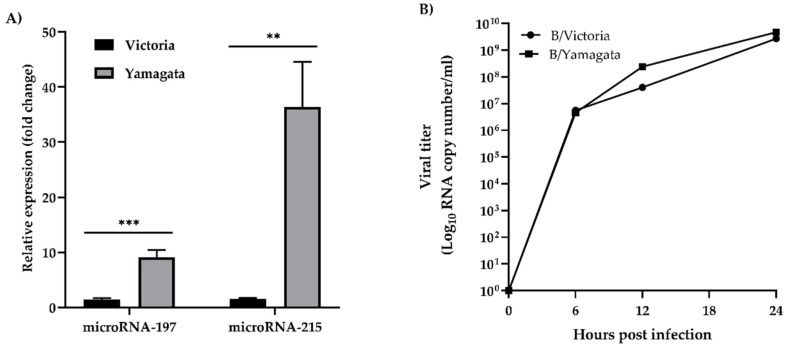
(**A**) Validation of cfa-miR-197 and cfa-miR-215 at 24 hpi by RT-qPCR. Although both cfa-miR-197 and cfa-miR-215 were upregulated upon influenza B virus infection, the expressions of both microRNAs found in the MDCK cells infected with Yamagata lineage were significantly higher than those found in Victoria lineage infection (*p* ≤ 0.01 is designated as **; *p* ≤ 0.001 is designated as ***). RNU6-2 was used as an internal control and mock-infected cells were used as a calibrator sample. (**B**) Viral replication of influenza B viruses, Victoria and Yamagata lineages. Viral titers were determined at 6, 12, and 24 hpi by RT-qPCR.

According to previous studies [21,22], two out of five microRNAs including cfa-miR-197 and cfa-miR-215 are involved in virus infections. Therefore, these two microRNAs were selected for further validation. To confirm the results obtained from high-throughput data, the expression of both microRNAs was confirmed by using RT-qPCR (Figure 2A). The results showed that both microRNAs were upregulated upon infection of either Victoria or Yamagata lineage, which were consistent with the profiles obtained from Next-Generation Sequencing. However, the cells infected with Yamagata lineage significantly expressed higher levels of cfa-miR-197 (*p* = 0.0005) and cfa-miR-215 (*p* = 0.0018) than those infected with Victoria lineage. Meanwhile, the supernatants were collected at 6, 12, and 24 hpi in order to determine viral titer during influenza B virus infection (Figure 2B). The result showed that the replications of Yamagata lineage were 5.95-fold and 1.75-fold greater than that of Victoria lineage at 12 and 24 hpi, respectively. 

### 3.4. Karyopherin Alpha 6 (KPNA6) as a Target of cfa-miR-197

In order to predict the target genes which might be controlled by cfa-miR-197 and cfa-miR-215, three free-accessible computational programs including miRDB, TargetScan, and RNAhybrid were utilized in this study. Interestingly, the result demonstrated that KPNA6 could be targeted by cfa-miR-197 and cfa-miR-215. In addition, the binding sites and the hybridization patterns between the 3′-UTR of the KPNA6 mRNA and the seed regions of both microRNAs are shown in Table 4.

Cfa-miR-197 could target the 3′-UTR of the KPNA6 transcript at the position of 361–367 (5′-seed; MFE = −21.7 kcal/mol) and 4399–4405 (5′ canonical; MFE = −27.3 kcal/mol), whereas cfa-miR-215 could bind to KPNA6 at the position of 1169–1176 (5′ canonical; MFE = −22.7 kcal/mol) and 2784–2790 (5′-seed; MFE = −20.1 kcal/mol).

**Figure 3:**
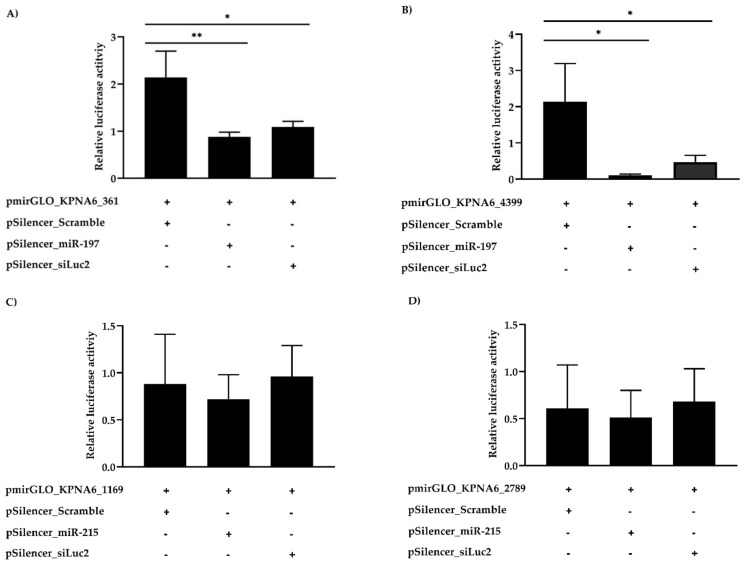
Luciferase activity. The MDCK cells were co-transfected with a reporter vector containing the target site of cfa-miR-197 and cfa-miR-215 (designated as pmirGLO_KPNA6) and a silencing vector encoding for scramble, cfa-miR-197, cfa-miR-215, siLuc2 (designated as pSilencer). (**A**) To determine whether the 3′-UTR position at 361–367 of the KPNA6 transcript was a target of cfa-miR-197. (**B**) To determine whether the 3′-UTR position at 4399–4405 of the KPNA6 transcript was a target of cfa-miR-197. (**C**) To determine whether the 3′-UTR position at 1169–1176 of the KPNA6 transcript was a target of cfa-miR-215. (**D**) To determine whether the 3′-UTR position at 2789–2790 of the KPNA6 transcript was a target of cfa-miR-215 (*p* ≤ 0.05 is designated as *; *p* ≤ 0.01 is designated as **).

To confirm whether KPNA6 was a putative target of cfa-miR-197 and cfa-miR-215 in MDCK cells, luciferase reporter assays were conducted at 48 h after co-transfection of pmirGLO containing the 3′-UTR of KPNA6 and pSilencer encoding microRNA mimic. As shown in Figure 3A,B, cfa-miR-197 inhibited luciferase activity in the cells transfected with pmirGLO containing the 3′-UTR of KPNA6 at both position of 361–367 and 4399–4405, respectively. However, cfa-miR-215 had no effect on luciferase activity in the cells transfected with pmirGLO containing the position of 1169–1176 or 2784–2790 (Figure 3C,D, respectively). 

**Figure 4:**
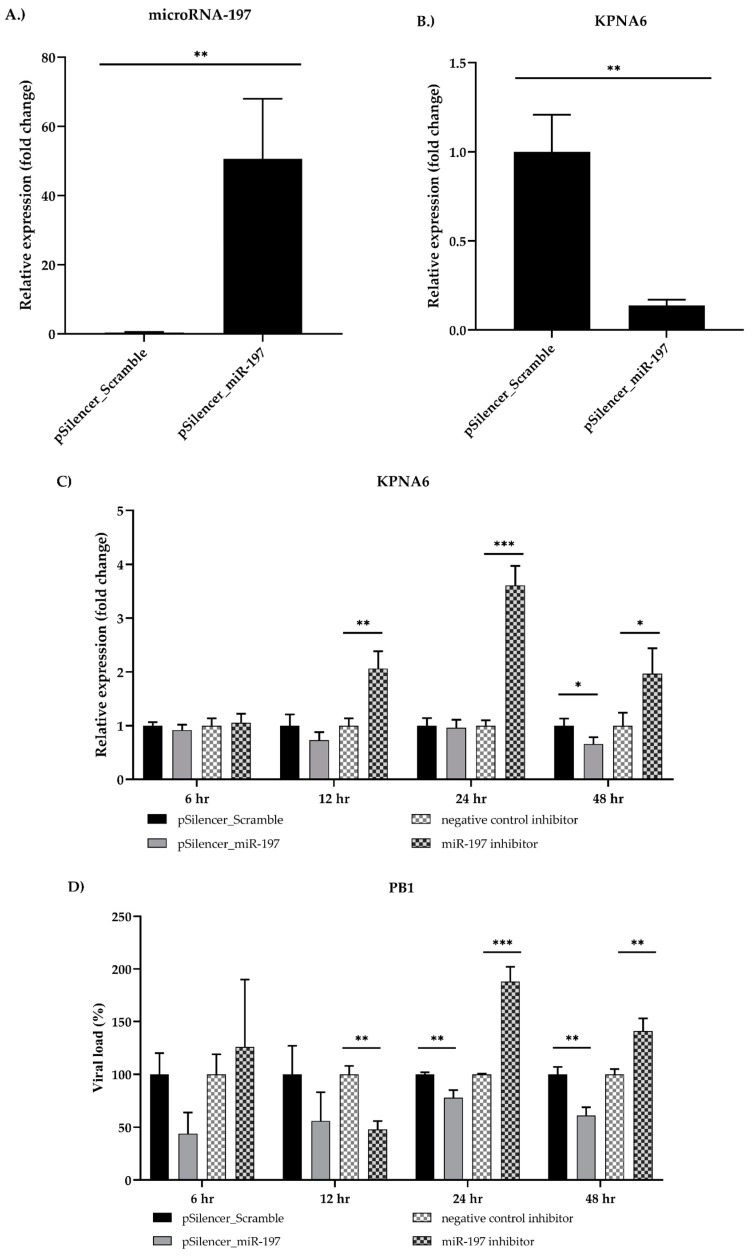
The effect of cfa-miR-197 on the expressions of KPNA6 mRNA and viral replication. The MDCK cells were overexpressed with pSilencer_miR-197, which encodes cfa-miR-197. After incubation for 48 h, the cells were collected. (**A**) cfa-miR-197 and (**B**) KPNA6 expression levels were determined in the cells transfected with pSilencer_Scramble or pSilencer_miR-197 for 48 h by RT-qPCR (*p* ≤ 0.01 is designated as **). Moreover, cfa-miR-197 was either overexpressed or inhibited, followed by infection of B/Victoria lineage viruses for 6, 12, 24, and 48 h. (**C**) Cellular KPNA6 in infected cells and (**D**) viral PB1 in supernatants were determined in the cells with pSilencer_miR-197 and miR-197 inhibitor by RT-qPCR (*p* ≤ 0.05 is designated as *; *p* ≤ 0.01 is designated as **; *p* ≤ 0.001 is designated as ***). RNA U6 and GAPDH was used as an internal control for microRNA-197 and KPNA6, respectively, whereas the cells treated with scramble and negative control inhibitor were used as calibrators for silencing vector and microRNA inhibitor, respectively.

Furthermore, the effect of cfa-miR-197 overexpression on KPNA6 expression was determined. This investigation demonstrated that cfa-miR-197 were overexpressed when the cells were treated with pSilencer_miR-197 (Figure 4A; *p* = 0.0074). Concurrently, upregulated cfa-miR-197 reduced KPNA6 expression on mRNA level (Figure 4B; *p* = 0.0075). Additionally, the effect of cfa-miR-197 on viral replication was investigated. The cells were transfected with pSilencer-miR-197 and cfa-miR-197 inhibitor, followed by influenza B virus infection for multiple time points (Figure 4C,D). The results demonstrated that the cells treated with plasmid overexpressing cfa-miR-197 had significant down-expressions of KPNA6 at 48 hpi (*p* = 0.0323) and viral PB1 at 24 and 48 hpi (*p* = 0.076 and *p* = 0.034, respectively). In contrast, the cells treated with cfa-miR-197 inhibitor demonstrated significantly higher expression of KPNA6 at 24 and 48 hpi (*p* = 0.003 and *p* = 0.0347, respectively) and PB1 at 24 and 48 hpi (*p* = 0.0004 and *p* = 0.005, respectively). Taken together, these results indicated that KPNA6 might be directly regulated by cfa-miR-197, affecting influenza B virus replication. 

## 4. Discussion

Nowadays MDCK cells are known to be permissive to influenza virus infection and replication. As a result of that, MDCK cells are widely used for identification and diagnosis of influenza viruses from clinical specimens. In addition, this permissive cell line could be utilized to produce influenza vaccines. Moreover, in vitro study is a cost-effective and accessible infection model for investigating host–pathogen interactions. Recently, a great deal of evidence has suggested that microRNAs play crucial roles in post-transcriptional gene regulation, thus leading to controlling various biological processes within a cell. In this study, microRNA profile of permissive MDCK cells upon infection of seasonal influenza B viruses was investigated.

Recently, it has been evident that cellular microRNAs could influence viral replication and pathogenesis through virus-induced alterations in the host transcriptome. For instance, several microRNAs impair the production of interferons during hepatitis C virus infection [23,24,25]. In addition, apoptosis is regulated by some microRNAs response to West Nile virus [26] and respiratory syncytial virus [27]. More specifically, hsa-miR-146a inhibits the expression of TNF receptor associated factor 6 (TRAF6), affecting interferon production against enterovirus 71 [28], dengue virus [29], and Japanese encephalitis virus [30]. Interestingly, our result demonstrated that cfa-miR-146b were upregulated during infection of influenza B virus (Yamagata lineage). According to the microRNA database miRBase, the seed region of cfa-miR-146b is similar to that of hsa-miR-146a. Nevertheless, the function of cfa-miR-146b in the immune response to influenza B virus might be further explored. 

In the previous years, microRNAs’ response to infection of influenza A viruses has been extensively studied in human cells. Recent investigations suggested that some microRNAs modulated pro-apoptotic and anti-apoptotic effects during influenza A viruses infected in A549 cell line [31,32]. Furthermore, antiviral proteins produced from human lung epithelial cells and dendritic cells were controlled by several influenza A virus-mediated microRNAs [33,34,35]. In addition to human cells, few studies demonstrated in vivo and in vitro microRNA expression in dogs infected with canine influenza A viruses [36,37]. Nonetheless, canine microRNAs’ response to seasonal human influenza virus infection has not been reported yet. Intriguingly, this study showed that cfa-miR-29c were increased when the cells were infected with influenza B virus (Victoria lineage). Similarly, the infection of seasonal influenza A (H1N1) virus could induce the expression of hsa-miR-29c, leading to lowered NF-κB activity and decreased proinflammatory cytokines through up-regulation of A20 [38]. However, the effect of cfa-miR-29c on innate immunity against influenza B virus infection should be further investigated. 

On the other hand, microRNAs’ profile upon seasonal influenza B virus infection has been little studied. Our result demonstrated that upregulation of cfa-miR-183 and downregulation of cfa-miR-486 were expressed in influenza B (Victoria lineage) virus-infected MDCK cells. This finding is consistent with the previous report of human lung epithelial cells infected with the same virus lineage [39]. Furthermore, this study showed that the expression of cfa-miR-197 and cfa-miR-215 was increased when the cells were infected with either Victoria or Yamagata lineage. Previously, microRNA-197 and microRNA-215 were induced by virus infections, influencing gene expressions in their host organisms. Hsa-miR-197 was downregulated by enterovirus 71 (EV71) to maintain ras-related nuclear protein (RAN), leading to nuclear transport of viral proteins [21]. Moreover, hsa-miR-215 regulated the expression of tripartite motif-containing 22 (TRIM22), inhibiting NF-κB signaling, resulted in enhanced hepatitis C virus replication [22]. In addition, microRNA-197 and microRNA-215 were reported to modulate the mechanisms of program cell death in many kinds of cancers. The proapoptotic activity of lysine 63 deubiquitinase (CYLD) was inhibited due to overexpressed microRNA-197 in lung adenocarcinoma cells [40]. Additionally, X-chromosome-linked inhibitor of apoptosis (XIAP) could be targeted by microRNA-215 [41,42]. Therefore, it might be feasible that both microRNAs are involved in influenza virus-mediated apoptosis. 

In this study, the gene targets of cfa-miR-197 and cfa-miR-215 were in silico predicted by three databases: miRDB, TargetScan, and RNAhybrid. It is intriguing that karyopherin alpha 6 (KPNA6) or importin-α7 could be co-targeted by both microRNAs. Nevertheless, luciferase activity revealed that KPNA6 contained the putative binding sites for only cfa-miR-197, but not cfa-miR-215. In addition, the downregulation of KPNA6 mRNA occurred after cfa-miR-197 was overexpressed. There is growing evidence that KPNA6 plays various roles in the pathogenesis and replication of many viruses such as Zika virus [43], Ebola virus [44,45], and porcine reproductive and respiratory syndrome virus [43]. In addition, the role of KPNA6 has been studied in influenza A viruses, which are more closely related to seasonal influenza B viruses. Recent studies indicated that KPNA6 interacts with some influenza A viral proteins, affecting viral replication and pathogenicity. Viral RNA polymerase basic 2 (PB2) is considered a major virulent determinant of influenza A viruses, which involves in host adaptation [46]. In mammalian-adapted strains, PB2 is imported into the host nucleus via preferential binding to KPNA6 [47,48]. Besides interspecies transmission, PB2 is thoroughly known to play a key part in the replication and transcription of influenza viruses. Therefore, KPNA6 is also essential for propagation of influenza A viruses [49,50,51,52]. Furthermore, viral nucleoprotein (NP) is another important multifunctional protein interacting with KPNA6, leading to enhancing influenza A virus replication [46,53]. A recent study has shown that a long N-terminal tail of the influenza B virus NP bound to human KPNA6 [54]. Although the NP of influenza B viruses could possibly be imported into canine nucleus by KPNA6, the interaction between NP of influenza B virus and canine KPNA6 should be experimentally investigated. Moreover, KPNA6 was shown to interact with not only viral proteins, but also host proteins [55]. For example, KPNA6 mediates Kelch-like ECH-associated protein 1 (Keap1)/Nuclear factor erythroid 2–related factor 2 (Nrf2) signaling pathway, affecting IFNα antiviral response, oxidative stress, and autophagy during hepatitis C virus infection [56,57].

In conclusion, this investigation provides canine microRNA profiles of permissive MDCK cell line infected with two different lineages of influenza B viruses, which are responsible for seasonal epidemics. Five common microRNAs including cfa-miR-197, cfa-miR-215, cfa-miR361, cfa-miR-1841, and cfa-miR-1842 were upregulated in the MDCK cells infected with either Victoria or Yamagata lineage. Among these microRNAs, KPNA6 could be the direct target of cfa-miR-197. Therefore, virus-mediated microRNAs may indirectly affect viral replication and pathogenesis through silencing host genes.

## Figures and Tables

**Table 1:** Primers and PCR conditions.

Primers	Nucleotide Sequences (5′–3′)	PCR Conditions (40 Cycles)
Cfa-miR-361_F	TCAGAATCTCCAGGGGTAC	95 °C 15 s, 58 °C 30 s, 62 °C 30 s
Cfa-miR-197_F	ACCACCTTCTCCACCCAG	95 °C 15 s, 58 °C 30 s, 62 °C 30 s
Cfa-miR-215_F	TGACCTACGAATTGATAGACA	95 °C 15 s, 55 °C 30 s, 62 °C 30 s
Cfa- RNU6-2 _F	CTCGCTTCGGCAGCACA	95 °C 15 s, 55 °C 30 s, 62 °C 30 s
Cfa-miRNA-qPCR_R	TGCGGATAACAATTTCACACA	-
Cfa-KPNA6_F775	CCAAAGAGCCTAGTCCTCCA	95 °C 15 s, 64 °C 20 s, 80 °C 20 s
Cfa-KPNA6_R926	CTGCTGAGAGGTTCCAGAGG	
Cfa-GAPDH_F85	GTGAAGGTCGGAGTCAACGG	95 °C 15 s, 60 °C 20 s, 72 °C 20 s
Cfa-GAPDH_R191	TCAATGAAGGGGTCATTGATGG	
Flu B_PB1_F269	AGGCTTTGGATAGAATGGATGA	95 °C 15 s, 57 °C 20 s, 72 °C 30 s
Flu B_PB1_R385	AAGTCTGTCTCCCCTGGGTT	

**Table 2:** Oligonucleotides used for construction of silencing and reporter plasmids.

Oligonucleotides ^1^	Nucleotide Sequences (5′–3′)	Plasmids
Cfa-miR-197_TS	GATCCGCGGGTAGAGAGGGCAGTGGGAGGTAAGAGCTCTT CACCCTTCACCACCTTCTCCACCCAGCTTTTTTGGAAA	pSilencer 3.0-H1
Cfa-miR-197_BS	AGCTTTTCCAAAAAAGCTGGGTGGAGAAGGTGGTGAAGGG TGAAGAGCTCTTACCTCCCACTGCCCTCTCTACCCGCG	
Cfa-miR-215_TS	GATCCATGACCTACGAATTGATAGACAATTTGGCTAAGTTT GTCTGTCATTTTTGTAGGCCATTTTTTGGAAA	pSilencer 3.0-H1
Cfa-miR-215_BS	AGCTTTTCCAAAAAATGGCCTACAAAAATGACAGACAAAC TTAGCCAAATTGTCTATCAATTCGTAGGTCATG	
Cfa-miR-Scramble_TS	GATCCGCAGGTCTTTCATCTAGAACGATGCGGGTTCAAGAG ACCCGCATCGTTCTAGATGAAAGACCTGTTTTTTGGAAA	pSilencer 3.0-H1
Cfa-miR-Scramble_BS	AGCTTTTCCAAAAAACAGGTCTTTCATCTAGAACGATGCGG GTCTCTTGAACCCGCATCGTTCTAGATGAAAGACCTGCG	
siLuc/Luc2_TS	GATCCCACCCCAACATCTTCGACGTTCAAGAGACGTCGAAG ATGTTGGGGTGTTTTTTGGAAA	pSilencer 3.0-H1
siLuc/Luc2_BS	AGCTTTTCCAAAAAACACCCCAACATCTTCGACGTCTCTTGA ACGTCGAAGATGTTGGGGTGG	
KPNA6_361_TS	CTAGTTATTTTTTTCTTTAGTGGTGACT	pmirGLO
KPNA6_361_BS	TCGAAGTCACCACTAAAGAAAAAAATAA	
KPNA6_4399_TS	CTAGGCTGTGCCGTGGGGCTGGTGAAGA	pmirGLO
KPNA6_4399_BS	TCGATCTTCACCAGCCCCACGGCACAGC	
KPNA6_1169_TS	CTAGATTCTATATATTAGGTAGGTCAAT	pmirGLO
KPNA6_1169_BS	TCGAATTGACCTACCTAATATATAGAAT	
KPNA6_2784_TS	CTAGACCCTGGCTTCGATGAGGTCAAAG	pmirGLO
KPNA6_2784_BS	TCGACTTTGACCTCATCGAAGCCAGGGT	

^1^ Abbreviations: TS: Top Strand; BS: Bottom Strand.

**Table 3:** The number of predicted microRNA target genes in response to influenza B viruses.

Biological Processes ^1^	The Number and the Percentage of microRNA Target Genes ^2^				
	Cfa-miR-197	Cfa-miR-215	Cfa-miR-361	Cfa-miR-1841	Cfa-miR-1842
BA	3 (1.95%)	4 (4.08%)	3 (1.58%)	10 (1.72%)	5 (1.49%)
BP	0 (0%)	0 (0%)	1 (0.53%)	0 (0%)	0 (0%)
BR	33 (21.43%)	20 (20.41%)	38 (20.00%)	111 (19.07%)	56 (16.67%)
Pro	1 (0.65%)	0 (0%)	2 (1.05%)	2 (0.34%)	1 (0.30%)
CC	2 (1.30%)	0 (0%)	0 (0%)	5 (0.86%)	4 (1.19%)
CP	46 (29.87%)	31 (31.63%)	56 (29.47%)	186 (31.96%)	94 (27.98%)
DP	3 (1.95%)	2 (2.04%)	3 (1.58%)	15 (2.58%)	12 (3.57%)
IS	2 (1.30%)	1 (1.02%)	2 (1.05%)	5 (0.86%)	6 (1.79%)
Lo	20 (12.99%)	12 (12.24%)	21 (11.05%)	62 (10.65%)	35 (10.42%)
MP	33 (21.43%)	17 (17.35%)	47 (24.74%)	156 (26.80%)	84 (25.00%)
MO	5 (3.25%)	6 (6.12%)	14 (7.37%)	29 (4.98%)	23 (6.85%)
Pi	0 (0%)	0 (0%)	1 (0.53%)	0 (0%)	0 (0%)
Re	0 (0%)	0 (0%)	5 (2.63%)	3 (0.52%)	5 (1.49%)
RS	5 (3.25%)	3 (3.06%)	7 (3.68%)	18 (3.09%)	11 (3.27%)
RP	1 (0.65%)	0 (0%)	0 (0%)	0 (0%)	0 (0%)
Si	0 (0%)	0 (0%)	0 (0%)	0 (0%)	1 (0.30%)
**Total genes**	**154**	**98**	**190**	**582**	**336**

^1^ GO biological processes of target genes were categorized by PANTHER classification system version 14.1 as follows: BA: biological adhesion (GO:0022610); BP: biological phase (GO:0044848); BR: biological regulation (GO:0065007); Pro: cell proliferation (GO:0008283); CC: cellular component organization or biogenesis (GO:0071840); CP: cellular process (GO:0009987); DP: developmental process (GO:0032502); IS: immune system process (GO:0002378); Lo: localization (GO:0051179); MP: metabolic process (GO:0008152); MO: multicellular organismal process (GO:0032501); Pi: pigmentation (GO:0043473); Re: reproduction (GO:0000003); RS: response to stimulus (GO:0050896); RP: rhythmic process (GO:0048511); Si: signaling (GO:0023052). ^2^ The data collected from miRDB were analyzed as of June 2019.

**Table 4:** Target site prediction and hybridization pattern of Karyopherin Alpha 6 (KPNA6)-microRNAs.

MicroRNAs	Position on 3′ UTR	Predicted Consequential Pairing between Target Region (top) and microRNA (bottom)	MFE (kcal/mol)
Cfa-miR-197	361–367	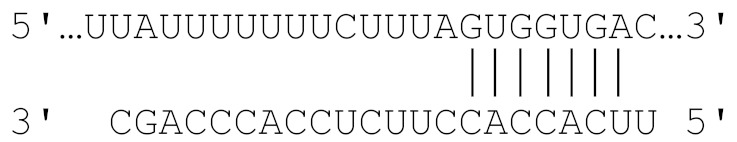	−21.7
	4399–4405	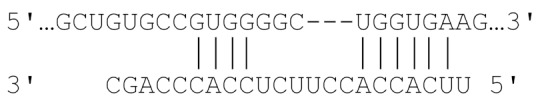	−27.3
Cfa-miR-215	1169–1176	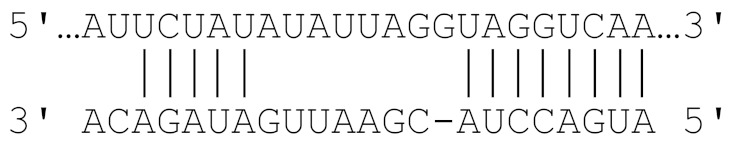	−22.7
	2784–2790	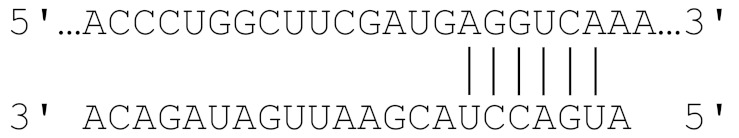	−20.1

Note: The data were analyzed by TargetScan version 7.2, miRDB, and RNAhybrid as of June 2019.
